# Inflammation and cancer: molecular mechanisms and clinical consequences

**DOI:** 10.3389/fonc.2025.1564572

**Published:** 2025-03-17

**Authors:** Hikmet Akkız, Halis Şimşek, Deniz Balcı, Yakup Ülger, Engin Onan, Nevin Akçaer, Anıl Delik

**Affiliations:** ^1^ Department of Gastroenterology, Medical Faculty, Bahçeşehir University, İstanbul, Türkiye; ^2^ Department of Gastroenterology, Medical Faculty, Hacettepe University, Ankara, Türkiye; ^3^ Department of Gastroenterology, Medical Faculty, Cukurova University, Adana, Türkiye; ^4^ Department of Nephrology, Medical Faculty, Baskent University, Adana, Türkiye; ^5^ Department of Gastroenterology, Medical Faculty, Health Sciences University, Adana, Türkiye; ^6^ Department of Biology, Science and Literature Faculty, Cukurova University, Adana, Türkiye

**Keywords:** tumor microenvironment, cancer-associated inflammation, cancer-associated fibroblasts, gastro-intestinal cancer, innate immunity, adaptive immunity

## Abstract

Inflammation, a hallmark of cancer, has been associated with tumor progression, transition into malignant phenotype and efficacy of anticancer treatments in cancer. It affects all stages of cancer, from the initiation of carcinogenesis to metastasis. Chronic inflammation induces immunosup-pression, providing an environment conducive to carcinogenesis, whereas acute inflammation induces an antitumor immune response, leading to tumor suppression. Solid tumors have an inflammatory tumor microenvironment (TME) containing cancer cells, immune cells, stromal cells, and soluble molecules, which plays a key role in tumor progression and therapy response. Both cancer cells and stromal cells in the TME are highly plastic and constantly change their phenotypic and functional properties. Cancer-associated inflammation, the majority of which consists of innate immune cells, plays an important role in cancer cell plasticity, cancer progression and the development of anticancer drug resistance. Today, with the combined used of advanced technologies, such as single-cell RNA sequencing and spatial molecular imaging analysis, the pathways linking chronic inflammation to cancer have been largely elucidated. In this review article, we highlighted the molecular and cellular mechanisms involved in cancer-associated inflammation and its effects on cancer progression and treatment response. We also comprehensively review the mechanisms linking chronic inflammation to cancer in the setting of GI cancers.

## 1 Introduction

Inflammation is an evolutionary process involving the recruitment, activation and action innate and adaptive immune cells (1–4). In addition to its role in host defense against pathogens, inflammation plays a critical role in tissue repair, regeneration, and remodeling, and mild inflammation is necessary to maintain tissue homeostasis (5–8). The canonical inflammatory process is characterized by a series of vascular changes, the release of inflammatory mediators, and recruitment of inflammatory immune cells in inflammatory sites (8–11). In addition to developing in tissue damage and infection, chronic inflammation also occurs in other serious diseases, such as diabetes, atherosclerosis, and cancer (10, 11). The association between cancer and inflammation has been known for a long time. In 1863, the German pathologist Rudolph Virchow observed the presence of inflammatory infiltrates in solid tumors and hypothesized that cancer develops at sites of chronic inflammation (12). Around the same time, William Cooley, pioneer of cancer immunotherapy, showed that some patients displayed tumor regression after being injected with immune stimulatory Streptococcus pyogenes cultures (8, 10). In solid tumors, including gastrointestinal (GI) cancers, molecular features of cancer cell, such as genetic aberrations, epigenetic modifications, signaling pathway deregulation and high metabolic stress, play key roles in shaping an inflammatory tumor microenvironment (TME) that is a major determinant of the biological behavior of tumor cells, and thus tumor progression and clinical outcome (1, 8, 11, 13–17). Inflammation, regardless of diseases from which it originates, has an important effect on the formation of the cellular composition of the TME (8, 10, 11, 17).

During cancer progression, cancer cells develop strategies to evade immune surveillance, such as downregulation of antigen presentation mechanism and induction of immune checkpoint molecules (5, 6, 8, 10, 11, 17). Concurrently, cancer cells hijack immune cells such as neutrophils, macrophages and regulatory T cells (Treg) to regulate an inflammatory TME that promotes immune escape (5, 6, 8, 10, 11, 17). Cancer-associated inflammation is the chronic inflammatory component of the TME and is emerged at all stages of tumor from the onset of carcinogenesis to advanced stage (8, 11, 17–21). It plays a key role in the recruitment of innate immune cells, such as macrophages and neutrophils, and immunosuppressive cells, such as myeloid-derived immunosuppressive cells (MDSCs) and regulatory T (Tregs) cells in the TME, contributing significantly to the shapping the inflammatory and immunosuppressive TME (1, 19, 21). Cancer-associated inflammation plays a critical role in the plasticity of cancer cells and stromal cells as well as shapping the cellular composition of the TME (8, 11, 16, 17, 21, 22). Additionally, it may also contribute to the recruitment of oncogenic mutations and predispose to the development of metastatic lesions (21, 22). Because of all these impacts, cancer-associated inflammation is an important driver of the malignant biological behavior of the tumor (1, 8, 10, 11, 17, 21, 22). Therapy-induced inflammation, which occurs in response to various anti-cancer therapies, including chemotherapy, radiotherapy, and immunotherapy, promotes drug resistance and cancer progression (8–11). Acute inflammation in solid tumors caused by various factors displays anti-cancer function through inducing the activation of dendritic cells (DCs) and CD8^+^ T cells (1, 8, 10, 11). Unlike the inflammatory response following infection and tissue injury, cancer-associated inflammation is unresolved in character (19–21). Additionally, cancer-extrinsic inflammation induced by environmental factors, such as obesity, smoking, and excessive alcohol consumption has been shown to increases cancer risk and accelerates tumor progression (8, 10, 11). In this review article, we focus on the molecular and cellular mechanisms involved in the pathogenesis of cancer-associated inflammation, as well as the dynamic and complex interactions between cancer-associated inflammation, cancer cells, and immune system. Understanding all aspects of this crosstalk will pave the way for the way for the development of more effective molecular targeted therapies for cancer treatment.

## 2 Initiation of inflammation and general description

Inflammation is a fundamental immune response that follows tissue injury and infection (10, 11). Many soluble molecules released from damaged tissues and activated immune cells participate in the inflammatory response, such as cytokines, chemokines and growth factors (8, 10, 11). The initial phase of inflammation is triggered by an inter-action between pattern recognition receptors (PRRs) and pathogen-associated molecular patterns (PAMPs) (8, 10, 11). In this stage, PAMPs are recognized by tissue macrophages or other innate immune cells, such as neutrophils, and dendritic cells (DCs), promoting the expression of pro-inflammatory mediators, accentuating the immune response (10, 11, 22). Additionally, damaged tissue secretes a variety of signaling molecules, such as cytokines, chemokines and adhesion molecules, leading to the accumulation of innate immune cells in the inflammatory field (3, 8, 10, 22–26). In the second stage of the inflammatory cascade, inflammatory cells undergo apoptosis and are phagocyted by macrophages (11, 27–30). Tissue-resident macrophages respond to tissue changes by several mechanisms, such as eliminating dying cells, expressing chemotactic molecules, orchestrating immune mechanisms, and contributing to the recruitment of stem cells (8, 10, 29). In the first two stages of inflammatory cascade, inflammation exerts a significant immunostimulatory effects (8, 10, 11). The resolution phase, which is the 3rd phase of inflammation cascade, is characterized by the secretion of anti-inflammatory mediators, such as specialized proresolving lipid mediators (SPMs, e.g., lipoxin A4 (LXA4) and resolving D1 (RVD1), cytokines such as IL-10 and growth factors including TGFβ and epidermal growth factor (EGF) (3, 8, 9, 11). SPMs play an important role in the resolution of inflammation by impeding neutrophil recruitment in damaged tissue, attenuating the secretion of inflammatory cytokines, and fostering the capacity of macrophages to phagocyte apoptotic neutrophils (30–33). These mediators augment the production of Tregs and B cells to suppress excessive activation of adaptive immunity (30, 32). In the final phase of the inflammatory cascade, the tissue repair process modifies the inflammatory process, weakens the inflammatory responses, and rebuilds tissue homeostasis (3, 8, 10, 11).

Inflammation can be defined as acute and chronic inflammation depending on the duration of the disease (8, 10, 11). Acute inflammation is the initial response to infection or tissue injury; the majority of cells involved in acute inflammation being granulocytes (11). Chronic inflammation is one of the main drivers in the development and progression of cancer (3, 8, 10, 11). Clinical and experimental studies have shown that activation of inflammatory pathways leads to destructive inflammation in the TME, which causes phenotypic and functional changes contributing to cancer progression (8, 10, 22, 34). The immune cell types and mediators secreted by them that participate in chronic inflammation are quite different from those in acute inflammation (1, 7, 8, 10, 35). The majority of cells involved in chronic inflammation belong to the adaptive arm of immune system (11, 22). In contrast to the acute inflammatory response, chronic inflammation is typically activated by DAMPs in the absence of activation of PAMPs (8, 10, 22). Chronic inflammation is regulated by specific signaling pathways that act as suppressors or activators (24–26). Molecular features of cancer cells, such as activation of oncogenes, inactivation of tumor suppressor genes and epigenetic modifications, promote the activation of various transcription factors in the TME, such as NF-κB, STAT3, and HIF-1α, which induce the production soluble molecules by the cancer cells, creating an inflammatory TME (8, 9, 11, 35). NF-κB activated by oxidative stress and pro-inflammatory cytokines initiates inflammation-associated cellular transformation through the expression of various genes, including anti-apoptotic proteins (BCL-XL, BCL-2), cytokines (TNF-α, IL-1β, IL-6, IL-8), inflammatory enzymes (iNOS, and COX-2), active molecules in metastasis, such as adhesion molecules and matrix metalloproteases (MMPs), cell cycle molecules (c-MYC and cylin D1), and angiogenic factors (VEGF and angiopoetin) (11, 35). Therefore, inflammation plays a critical role in all stages of cancer progression (11, 35). Several studies have shown that the combination chemotherapy with anti-inflammatory therapy has a favorable effect on treatment responses and patient survival (11, 35).

## 3 Molecular mechanisms linking chronic inflammation to cancer in the setting of GI cancers

Epidemiological studies have demonstrated that inflammation is closely related to cancer initiation and development. Approximately 25% of cancers arise from a chronic inflammatory microenvironment (1, 3, 8, 10, 11). Gastrointestinal (GI) cancers, such as colorectal cancer (CRC), stomach cancer, pancreatic cancer and liver cancer, are a leading cause of new cancer cases and cancer-related death, representing 26% of the global cancer incidence and 35% of all cancer-related deaths (1).

### 3.1 Gastric cancer-inducing inflammation

Gastric cancer is a major global health problem with >1,1 million new cases and >750,000 deaths each year (36). Gastric cancer is a prime example of chronic inflammation-associated cancer, which usually develops from chronic gastritis (36). Gastric cancer has two major subtypes: diffuse and intestinal type (37). Intestinal-type gastric carcinoma is more common than diffuse-type tumors and its main cause is Helicobacter pylori infection (37). Helicobacter pylori infection causes chronic gastritis that is associated with the generation of reactive oxygen species (ROS) and nitric oxide metabolites and a reduction of vitamin C levels, which can lead to peptic ulcer, gastric cancer, and gastric mucosa-associated lymphoid tissue lymphoma (17, 36, 38). In Asian and Eastern European countries, where gastric cancer incidence and mortality are high, the lifetime risk of developing gastric cancer in H. pylori-positive individuals is 1-5% (17). Additionally, in these geographic regions, high salt intake, smoking habits, low iron levels, and pickled foods contribute to the development of gastric cancer (17). Chronic inflammation damages parietal cells and these cells lose their acid-producing properties, resulting in hypochlorhydria or achlorhydria (17). The hypochlorhydric stomach creates a favorable microenvironment for the colonization of proinflammatory microorganisms, leading to the production of genotoxic metabolites and carcinogens, which are directly effective on malignant epithelial cell transformation (17, 37, 38). The etiology of diffuse-type gastric cancer remains unclear, although its incidence continues to increase globally (36).

### 3.2 Pancreatic cancer-driving inflammation

Pancreatic ductal adenocarcinoma (PDAC) is one of the most lethal cancers, with a 5-year survival rate of below 10% (39). Stromal desmoplasia and persistent activation of the immune system are the main features of PDAC and play a key role in cancer initiation and progression (3, 39). Inflammation, in which activated immune cells secrete a variety of proinflammatory molecules, plays an important role in pancreatic carcinogenesis (3, 39, 40). The continuous interaction between progenitor cells and innate and adaptive immune cells promotes cancer initiation by converting normal progenitor cells into cancer stem cells (3, 40). During this interaction, soluble molecules secreted by immune cells regulate cell migration, proliferation and survival (3, 39, 40). Pancreatic intra-epithelial neoplasia (PanIN) is developed through different stages of PDAC (3, 40). The PanIN lesion microenvironment contains macrophages, neutrophils and fibroblasts (3, 39, 40). As PanIN progresses, CXCL17-secreting macrophages and neutrophils recruit immature DCs (39, 40). Downregulation of CXCL17 develops immune tolerance toward tumor cells. IL-6 is essential for the maintenance of PanIN lesions and activates the MAPK and P13K/AKT pathways in these lesions (3, 41). Acinar cells with KRAS mutation have been indicated to trigger inflammation. Chronic inflammation may lead to PDAC in the absence of p53 (41). In a recently published study, researchers showed that upregulated expression of IKK2 or COX-2 in the absence of p53 leads to chronic inflammation-induced DNA mutations in the KRAS gene and PDAC via various signaling pathways (42). Chronic pancreatitis is the most common risk factor for PDAC, and the relative risk of PDAC in patients with chronic pancreatitis has been reported to be as high as 7.6-68.1 times (40). In studies conducted in Western countries, the incidence of PDAC in patients with chronic pancreatitis has been reported as 1.0-2.6% (40). PDAC has a highly stromal TME that contributes to its poor prognosis (39, 41).

### 3.3 Chronic inflammation-related colorectal cancer

**Figure 1:**
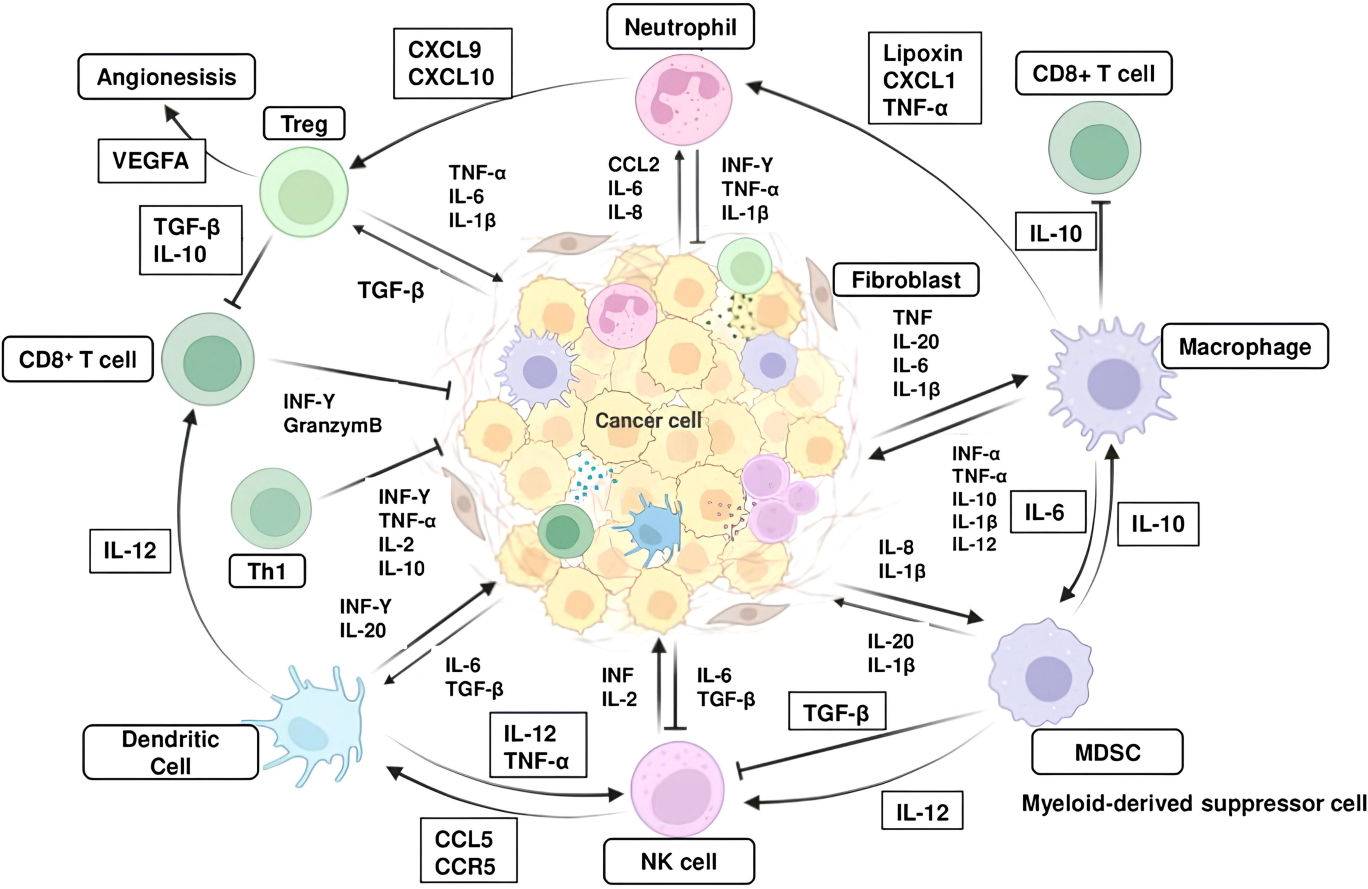
Interaction between inflammatory cells and inflammatory molecules in the tumor microenvironment. The major inflammatory cells include T helper cell (Th1), regulatory T cells (Tregs), Cytotoxic CD8^+^ T cells, macrophages, myeloid-derived suppressor cells (MDSCs), naturel killer (NK) cells and dendritic cells (DCs). Abbreviations: CXCR, CXC-Chemokine receptor; CXCL, Chemokine (C-X-C motif) ligand, TGF-β, transforming growth factor-β; TNF, tumor necrosis factor; IL, interleukin; IFN, interferon.

Another well-known example of chronic inflammation-derived cancer is colitis-associated colorectal cancer (CAC) (13, 14). Persistent chronic inflammation of the colon caused by inflammatory bowel diseases (IBDs), such as Crohn’s colitis and ulcerative colitis, is associated with an increased incidence of CAC (13, 14, 43, 44). The risk of CAC is high in patients with long-standing colitis, a large diseased colon segment and concomitant inflammatory diseases, such as primary sclerosing cholangitis (13, 14, 43, 44). Chronic inflammation itself is independent driver in the development of CAC in IBD (13, 14). Chronic inflammation-associated CAC is thought to arise as a result of the expansion of pro-tumorigenic clones (14). Somatic driver mutations can be detected in non-dysplastic inflamed colon years before the diagnosis of CAC (13). Multiple studies using whole exome sequencing have identified TCGA point mutation in the KRAS, BRAF, ERBB2, ERBB3, TP53, and FBXW7 genes in non-dysplastic colon mucosa (43). Local tissue damage leads to inflammation, which cooperates with driver mutations in the KRAS and p53 genes for malignant transformation of the cell (13, 43). Mutations may not be the initial event that triggers carcinogenesis in IBD (43). While p53 mutation is not detected in the colon mucosa, it is detected in approximately 50% of the dysplastic mucosa, and the frequency of p53 mutations gradually increases towards the dysplastic-carcinoma cascade (13, 44). P53 protein exerts transcriptional antagonism to NF-κB, a key regulator of inflammation, in cancer (13, 14, 43, 44). Additionally, copy number alterations accumulate progressively from low-grade dysplasia to high-grade dysplasia and cancer (13, 43, 44). Chatila et al. showed that cancer development in IBD predominantly arises from independent genetic events (14). Two important differences have been detected between CAC and sporadic CRC in terms of genomic landscapes (14). The first is that p53 alterations are early and highly recurrent events in CACs, which occur in half of dysplasia, while it is a late event in sporadic CRC (14). Secondly, while APC mutations are detected at a rate of 81% in sporadic microsatellite stable (MSS) CRC, they are detected at a rate of 11-22% in CAC (14). Studies in mouse models have demonstrated that SMAD4 is also an important player in the regulation of inflammation and loss of SMAD4 leads to the upregulation of some inflammatory signaling pathways, including IL-6/STAT3 and NF-κB (45). Many studies have shown that immunotherapy prevents cancer recurrence and improves overall survival in patients with MSI-H/dMMR (Microsatellite instability-high/mismatch re-pair-deficiency) colorectal cancer (46). **Figure 1**.

### 3.4 Link chronic inflammation and liver cancer

Chronic liver diseases are characterized by persistent inflammation and subsequent liver fibrosis, leading to liver cirrhosis and hepatocellular carcinoma (HCC) (47). HCC represents the most common type of liver cancer, which usually arises in the inflamed liver microenvironment caused by HBV and HCV infection, alcohol abuse and metabolic dysfunction-associated steatotic liver disease (MASLD) (47). MASLD is a spectrum of chronic liver disease that ranges from simple steatosis to metabolic dysfunction-associated steatohepatitis (MASH) and is strongly associated with metabolic syndrome (47). MASH is an emerging risk factor for HCC (47). Accumulating evidence has indicated that pre-cirrhotic MASLD might provide an increased risk of HCC, independent of cirrhosis (48). A range of single nucleotide polymorphisms, such as patatin-like phospholipase domain containing 3 (PNPLA3; rs 738409) and transmembrane superfamily member 2 (TM6SF2; rs 58542926) have been associated with the presence of MASLD and disease progression to advanced fibrosis and HCC (49, 50). Many research groups from different countries have shown that HCC is 3 times higher in patients carrying the PNPLA3 polymorphism (46, 49). Genomic analyses have indicated key pathways altered in HCC, including Wnt/β-catenin, P13K/Ras, and cell-cycle pathways (51, 52). The most frequently mutated genes in MASH-associated HCCs are TERT, CTNNB1, TP53 and ACVR2A genes (52). MASH-associated HCC samples have significantly higher rates of ACVR2A mutations than samples of other etiologies (52). The Wnt/TGF-β proliferation subclass is more frequent in MASH-driven HCC than in HCCs of other etiologies (53). Another molecular landscape of MASH-driven HCC is that it has an immunosuppressive pro-carcinogenic and inflammatory tumor microenvironment (52, 53).

In homeostasis, immune cells, particularly KCs are densely populated in the liver, which rapidly sense hepatocyte stress and injury signals, and lead to the activation of pro-inflammatory pathways (51). Metabolic stress induced by several factors causes metabolic disturbance in hepatocytes, increasing reactive oxygen species (ROS), endoplasmic reticulum (ER) stress and oxidative stress and resulting in hepatic metabolic reprogramming (47, 48, 51). These processes result in hepatocyte death of apoptotic or necroptotic type (48, 51). In the liver, dying or damaged hepatocytes release soluble mediators that act as damage-associated molecular patterns (DAMPs) (51). Preclinical studies have revealed that dying hepatocytes release P2Y14 ligands, such as uridine 5’-diphosphate (UDP)-glucose and UDP-galactose, that bind to the P2Y14 receptor on hepatic stellate cell (HSC) and induce activation in both mouse and human HSCs (54). Liver parenchymal and non-parenchymal cells, including HSCs, KCs, and liver sinusoidal endothelial cells (LSECs) perceive these dangerous signals released from the dying hepatocytes via PRRs and form inflammasome as the first response (55). Inflammasome, a protein complex, initiates the inflammatory response by producing IL-1β, releasing IL-18, and ultimately promoting inflammatory cell death (55). Pro-inflammatory cytokine-producing Innate and adaptive immune cells rapidly accumulate in the inflammatory microenvironment and disrupt hepatocyte metabolism by promoting hepatic metabolic reprogramming, thereby promoting hepatocyte injury and death, DNA damage, and hepatocyte proliferation (56). Inflammatory molecules promote the activation of HSCs, and inflammatory mediators secreted by activated HSCs contribute to the expansion of chronic inflammation and hepatocarcinogenesis (49, 51). Mounting evidence indicates that chronic liver inflammation damages hepatic epithelial cells, including hepatocytes and biliary epithelial cells (51). Simultaneously, chronic inflammation in-duces ROS production and DNA damage, increasing the frequency of genomic alterations (49). Furthermore, chronic liver inflammation induces phenotypic changes in hepatocytes and hepatic immune cells, especially macrophages (16, 49, 51). Chronic inflammation initiates hepatocarcinogenesis through the transformation of hepatocytes into liver progenitor cells and the differentiation of macrophages into tumor-associated macrophages (M2 phenotype) (16). TNFα and IL-6 produced by macrophages in the cirrhotic liver, as well as TNF receptor 1 (TNFR1) signaling expressed by hepatocytes play critical roles in the development and progression of HCC (16). TNFα is one of the protumorigenic cytokines, that activates both NF-κB and JNK signaling pathways (16, 51). Many studies demonstrate that activation of innate immune receptors, such as Toll-like 4 receptors (TLRs), plays a role in HCC development (16, 57).

The gut microbiome, which harbors more than 100 trillion microorganisms including bacteria, viruses, fungi and archaea, plays a critical role in the development and progression of HCC by contributing to the establishment and growth of chronic liver inflammation (58, 59). The liver regulates the intestinal microbiota with bile containing bile acids, IgA and antibacterial metabolites (60). Environmental factors, such as high-fat diets and alcohol consumption, can disrupt microbial compositions, leading to gut dysbiosis, which induces intestinal inflammation, which contributes to intestinal barrier dysfunction and translocation of microorganism-associated molecular patterns (MAMPs), such as lipopolysaccharide (LPS), to the liver and systemic circulation (61). In diet-induced NASH mouse models, a positive correlation between serum LPS levels and liver injury has been demonstrated (62). Cholestasis due to liver tissue remodeling in cirrhosis leads to intestinal dysbiosis (58). Cirrhosis patients display increased bacterial abundance in hepatic tissue, which induces pronounced transcriptional changes, including activation of fibro-inflammatory pathways as well as circuits mediating cancer immunosuppression (63). Increased intestinal permeability in cirrhotic patients allows the translocation of MAMPs and contributes to increased systemic and local inflammation in the liver (63). Dysbiosis changes the metabolism of intestinal bile acids, with less conversion of primary to secondary bile acids (64). Several studies have indicated that fecal microbial diversity is decreased in patients with cirrhosis compared with healthy controls, however, diversity increases as one progresses from cirrhosis to HCC (65). Preclinical studies have shown that MYC-transgenic mice capable of developing HCC arise lower numbers and sizes of HCC when given antibiotics (66). Another key finding of this study is that primary bile acids increase the accumulation of hepatic NK cells, whereas secondary bile acids reverse this situation (66). Several mouse model trials have revealed a link between the activation of inflammatory signals caused by intestinal permeability, the translocation of MAMPs, and the development of HCC (67). Rats with diethylnitrosamine-induced HCC have increased serum LPS, and administration of antibiotics decreased the tumor size and numbers (67). Another remarkable finding of this study was that tumor size was significantly reduced in TLR4-knock-out mice treated with diethylnitrosamine compared to wild-type mice (67).

## 4 Main drivers involved in the generation of cancer-association inflammation

Cancer-associated inflammation is characterized by the presence of inflammatory cells and inflammatory mediators, such as chemokines, cytokines and prostaglandins, tissue remodeling and angiogenesis in the TME (8–11, 18, 20, 22, 34, 35).

### 4.1 Genetic aberrations in oncogenes and tumor suppressor genes

The cancer genome somatic alterations, such as point mutations, genomic amplifications and rearrangements, play an important role in the development of cancer and in shaping of the inflammatory TME (18, 21). Tumor-derived cytokines and chemokines, tumor oncogenes and mutational burden determine the composition of the TME (18, 35). Emerging evidence has shown that there is a strong relationship between both tumor genotype/phenotype and immunological composition of the TME (35, 68). Oncogene-driven expression of cytokines critical for the recruitment and phenotype of immune cells, particularly cells of the myeloid lineage, has been reported (35). The effects of oncogenic pathways on the immune system, especially on inflammatory cells in cancer, vary according to the type, location and stage of cancer (69). KRAS mutations frequently occur in multiple cancers including CRC and PDAC, functioning as a ‘‘molecule switch’’ determining the activation of various oncogenic signaling pathways (35). In addition to its pro-tumorigenic role, KRAS also plays a key role in shaping an inflammatory and immunosuppressive TME through downstream effector activation and secreting various cytokines and chemokines (35). These soluble mediators promote the accumulation of suppressive immune cells into the TME while inhibiting the infiltration of T, B, and NK cells into the TME, thus facilitating unlimited proliferation of tumor cells. KRAS mutations can drive the secretion of anti-inflammatory cytokines, such as IL-10, TGF-β, and GM-CSF, as well as pro-inflammatory cytokines, such as ICAM-1, TNF-α, IL-1β, IL-6, and IL-18 (35, 69, 70). KRAS^G12D^ -driven PDAC secretes high levels of growth factor GM-CSF, which is associated with an increase in tumor-associated Gr+ CD11b+ myeloid cells and suppression of CD8+ T cells (71). Interestingly, genetic ablation of GM-CSF in mice results in decrease myeloid cell infiltration, improved CD8^+^ T cell infiltration into tumor, and substantially smaller lesion size (35). Another study found that KRAS^G12D^ expression is associated with infiltration of Treg cells in pancreatic cancer tissue (35, 72). In a mouse colorectal cancer model, KRAS^G12D^ expression inhibits T cell infiltration and interferon regulatory factor 2 (IRF2) production and, promotes the migration of CXCR2^+^ MDSCs into the TME (73). In experimental cancer models, multiple mutant KRAS variants led to increased IL-8, which promoted tumor-associated inflammation, angiogenesis, and tumor growth (21).

Another oncogene with strong immunoregulatory features is MYC, that play a critical role in proliferation, differentiation, metabolism and apoptosis (35, 74). Oncogenic MYC inhibits antitumor immunity by enhancing CD47 and PD-L1 expression to impair macrophage and T cell recruitment, production of IL-1β, and inhibiting the infiltration of CD8+ T cells, NK cells and DCs (21, 35). BRAF^V600E^ has been shown to induce Wnt/β-catenin signaling pathway, which in turn decrease production of CCL4, a chemokine important for the recruitment of CD103^+^ DCs (21). Additionally, BRAF^V600E^ has been revealed to promote production of factors such as IL-10 and IL-1α, which can induce tolerogenic forms of DC and CAFs (21). Notch signaling can activate monocytes and macrophages by driving CCL2 and IL-1β production, while promoting anti-tumor immunity by regulating TGFβ receptor and uPA production (18, 35, 74). Dysregulation of this pathway promotes epithelial-mesenchymal transition and angiogenesis (18, 35, 74). Oncogenic alterations in ERBB family members, including epidermal growth factor (EGF) and epidermal growth factor receptor 2 (EGFR2), promote cancer cell evasion of immune surveillance, thereby indirectly leading to an increase in the number of inflammatory cells in the TME (75). Mutant EGFR mediates cancer cell evasion of immune control by reducing PD-L1 expression, inhibiting CD8^+^ T cell recruitment, and promoting Treg infiltration (75). In GI cancers, human epidermal growth factor receptor (HER2) amplification downregulates MHC-1, promoting cancer cell immune evasion (76). Furthermore, HER2 amplification impedes antitumor immunity by inhibiting cGAS-STING pathway (76). Monoclonal antibodies targeting HER2, when combined with chemotherapy, improved patient survival with HER2^+^ cancer (77).

Inactivation or loss of tumor suppressor genes (TSGs) contributes significantly to cancer-associated inflammation (18, 35). P53 is a key regulator of cell cycle, DNA repair, senescence and apoptosis (78). Loss of p53 in cancer cells promotes secretion of WNT ligands, which in turn induces IL-1β production in TAMs in the TME. IL-1β enhanced accumulation of neutrophils, which accelerates tumor progression (79). In a KRAS^G12D^ -driven PDAC model, loss of p53 promoted tumor progression through increased expression of chemokines, such as CCL3, CCL11, CXCL5, and macrophage colony-stimulating factor (M-CSF), and the accumulation of macrophages and Treg cells within the TME (79). The p53 pathway can modulate the immunological composition of the tumor tissue by regulating NF-κB signaling, which is generally activated by the loss of p53 (8, 18, 21, 33, 35). NF-κB orchestrates cell survival and proliferation, but also the production of inflammatory cytokines (8, 9, 18, 21, 80). In experimental models, concomitant loss of E-cadherin and p53 promotes NF-κB activation, which is accompanied by increased macrophage recruitment and proinflammatory mediator production (21, 35, 80). Loss of p53 activates NF-κB, and induce the production of the inflammatory soluble molecules from cancer cells, which in turn alters the immune context through paracrine interactions (18, 80). Furthermore, studies in p53 knockout mouse models have revealed that NF-κB-mediated inflammatory response is a driving force of carcinogenesis (18, 80, 81). p53 missense mutations may enhance the malignant properties of cancer cells as well as the loss of p53 function (80, 82). Studies have shown that mutant p53 leads to the release of miR-1246-rich exomes, causing M1 macrophages to polarize into tumor-promoting M2 phenotype, thereby contributing to the establishment of an immunosuppressive and inflammatory TME (82–85). Loss of tumor suppressor gene LKB1 can promote the production of G-CSF, CXCL7, and IL-6, which induces neutrophil accumulation and can prevent the recruitment of immune cells that exhibit antitumor activity (18, 35). PTEN can inhibit NF-κB signaling, as such, loss of PTEN increases NF-κB-mediated expression of soluble molecules, which promotes the recruitment of inflammatory and immunosuppressive cells in the TME, such as neutrophil, macrophage, and Treg (81, 85). Furthermore, the mutational landscape of cancer cells, which directly reflects the immunogenicity of the tumor, may determine the extent and phenotype of the immune infiltrate, i.e. inflammation, in the TME (3, 18, 35, 80) **Figure 1**.

### 4.2 Crosstalk between tumor metabolism and inflammation

During tumor development and progression, cancer cells and their TME are continuously exposed to metabolic stress (86). To survive and growth, cellular adaptation and metabolic reprogramming are required (86). Tumor cells consume an enormous amount of glucose through enhanced aerobic glycolysis, resulting in decreased glucose levels in the tumor interstitial fluid (86). Aerobic glycolysis in cancer cells results in the production of large amounts of lactate that accumulate in the TME (35, 86). Lactate acts in an immunosuppressive manner in the TME and decreases cytotoxic activity in NK cells, and enhances proliferation, PD1 expression and the immunosuppressive capacity of Treg cells (1, 35, 86). Additionally, lactate increase MDSCs frequencies in the TME and induces an M2-like polarization in TAMs (1). In glucose deprivation, T cells may be forced to engage in strong oxidative stress, which is characterized by the production of reactive oxygen species (ROS) within cells, plays a critical role in the development of cancer and cancer-related inflammation (1, 35, 86). Excessive production of ROS can trigger chronic inflammation by activating the number of transcription factors such as NF-κB, AP-1, Wnt/β-catenin, p53, PPAR-γ, HIF-1α, and Nrf2 (86). The activation of these transcription factors leads to altered expression of various genes and proteins including growth factors, cell cycle regulatory molecules, oncogenes, tumor suppressor genes, pro-inflammatory cytokines, and chemokines (1, 82, 86). Lactate metabolism is a potential therapeutic target in GI tumors. Recent studies indicate that inhibition of lactate dehydrogenase A leads to the regression of tumor growth in preclinical models (86). The lipids can be taken up by immune cells, such as DCs and TAMs, resulting in enhanced lipid metabolism, high oxidative stress and ROS production (1, 86). Elevated levels of ROS are often associated with chronic inflammation (1, 86).

Tumor hypoxia develops as a result of the rapid consumption of oxygen by tumor cells and quick angiogenesis within the tumor (86, 87). Hypoxia leads to hypoxia-inducible factor (HIF)-1α induction, CAF and TAM activation, and the secretion of various chemokines, which in turn result in the accumulation of proinflammatory myeloid cells, particularly macrophages, in the TME (88). Hypoxia inhibits glycolysis and may promote the angiogenic activity of TAMs (86). In response to hypoxia, HIF-1α potentiates the polarization and suppression of the effect of M2 (89, 90). Hypoxia also promotes the progression of T cells to exhaustion status (90). It promotes the production of immune checkpoint molecules, such as PD1, PD-L1, CTLA-4, LAG-3, and TIM-3, contributing to the TME becoming more inflammatory and immunosuppressive (91). Although hypoxia affects all cells within the tumor tissue, two recent studies have shown that it specifically affects cells with an inflammatory phenotype (87, 88). Macrophages are effector immune cells that undergo significant changes when entering tumors or infected wounds (86–88). Hypoxia generates distinct responses from macrophages depending on the activation state (89). Hypoxia induces transcriptome turnover in macrophages, but inflammatory macrophages exhibit significantly increased mRNA destabilization compared to resting macrophages (87, 91, 92). In another study investigating the relationship between hypoxia and inflammation, Mello et al. found that hypoxia promotes the induction of inflammatory phenotype cancer-associated fibroblasts (iCAFs) by modulating their interaction with tumor cells and that hypoxic regulation of the iCAF phenotype is independent of tumor HIF1α (88). The drivers of tumor-associated inflammation are different in microbial-rich tumors and sterile tumors (8, 20). For example, in CRC, disruption of the intestinal barrier by oncogene in the mucosa where cancer originates leads to translocation of commensal bacteria and their metabolites, to promote IL-23 production and IL-23-mediated cancer-related inflammation (93, 94). In contrast, in tumors not originating in the mucosa genomic and metabolic changes, cell death and hypoxia may be initial inflammatory stimuli (94).

### 4.3 Key inflammatory cells and inflammatory molecules

Stromal cells, such as cancer-associated fibroblasts (CAFs), tumor-associated macrophages (TAMs), and tumor-associated neutrophils (TANs), are pivotal players in the generation and expansion of tumor-associated inflammation.

CAFs are one of the major components of the TME (1, 21, 95). Recent studies have demonstrated that CAFs display plasticity in response to cues from the TME and can have both tumor-promoting and tumor-limiting activities (1, 95). Studies using single-cell RNA sequencing broadly divided CAFs into 2 distinct subpopulations: inflammatory and growth factor-enriched CAF (iCAF) and myofibroblastic CAF (myCAF) (1, 95). Furthermore, additional CAF subpopulations have been identified in tumors such as HCC and CCA (1, 21, 95). CAFs critically modulate cancer progression through various mechanisms, including production of growth factors, inflammatory ligands and exosomes as well as ECM remodeling, angiogenesis, tumor mechanics and treatment responses (95). CAF-secreted inflammatory ligands and chemokines promote inflammation and tumor cell proliferation (8, 18, 95). Interaction between CAFs and cancer cells is mediated with a complex signaling network that consists of signaling pathways for TGFβ, mitogen-activated protein kinase (MAPK), Wnt/β-catenin pathway, JAK/STAT pathway, epidermal growth factor receptor (EGFR), and NF-κB (95). CAFs also secrete various molecules, such as platelet-derived growth factor (PDGF), human growth factor (HGF), vascular endothelial growth factor (VEGF), TNFα, and stromal cell-derived factor, to enhance tumor progression and inflammation (11, 21, 95). Additionally, these molecules reduce the tumor immunosurveillance and chemotherapy activity of drugs. During carcinogenesis, experimental trials have reported that cancer-derived CAFs modulate immune system through recruiting immune cells, such as neutrophils, monocytes and dendritic cells, and promote these cells to acquire immunosuppressive phenotypes that boost immune evasion (11, 95). The majority of ECM components expressed in the TME are produced by activated CAFs, which provides mechanical stability to the tumor (1, 95). Currently, CAF-targeted therapies aim to specifically deplete CAFs, impede their inflammation-promoting and immunosuppressive effects, or reprogram CAFs to a more quiescent state (1, 95).

Macrophages exert multifaced roles in cancer, a reflection of their plasticity in response to environmental stimuli (96, 97). Macrophages can be divided into two subtypes: proinflammatory M1 and anti-inflammatory M2 macrophages (97, 98). Tumor-associated macrophages (TAMs) are an important component of the TME and have an important role in the regulating of inflammation, angiogenesis, ECM remodeling, cancer cell proliferation, metastasis and immunosuppression and as well as resistance to cancer therapy (1, 2, 7, 18, 21, 98). M1 TAMs can be activated by TNF-α and GM-CSF and promote the recruitment and antitumor activities of CD8^+^ T cells and NK cells (98, 99). In the inflammatory TME, macrophages account for 30%-50% of cell populations (98–100). M1 TAMs exert a pro-inflammatory effect and play a critical role in the development and progression of tumors by expressing high levels of pro-inflammatory mediators, such as such as TNF-α, IL-1β, IL-6, IL-12, CXCL9 (98, 100, 101). TNF-α expression is increased aberrantly in many tumors, such as liver, breast and ovarian cancer, suggesting this cytokine is an important player in resistance to anticancer treatment (98). TNF-α exerts its antitumor effect by promoting apoptosis of tumor cells, polarizing TAMs to the M1 phenotype, and promoting EMT of tumor cells (102, 103). TNF-α displays its immunosuppressive effect by promoting the survival and function of Tregs (98). IL-6 triggers tumor progression by promoting tumor cell proliferation, survival, EMT, angiogenesis, and chemoresistance (98). In the early stage of cancer, proinflammatory cytokines, such as IL-6, IL-8, and TGF-β secreted from cancer cells, immune cells and stromal cells promote the recruitment of macrophages in the inflammatory TME and their polarization to the M2 phenotype (99, 100, 102–105). M1 macrophages kill tumor cells by secreting cytotoxic molecules, such as ROS and NO, or by antibody-dependent cell-mediated cytotoxicity (ADCC) (1, 98). In contrast, M2 macrophages are protumoral cells and function to suppress the activities of immune effector cells (98). For tumor healing, the proinflammatory M1 macrophages repolarize into anti-inflammatory M2 TAMs to control inflammation. M1 TAMs promote tumor-associated inflammation by producing pro-inflammatory mediators (2, 4, 7, 8, 10, 11, 14, 18, 19, 96, 97). M2 TAMs increase angiogenesis by upregulating angiogenesis-associated genes such as VEGF, PDGF, and PGE2, or via molecules CXCL12, IL-1β, IL-8 and Sema4d, leading to tumor progression (98, 102).

Neutrophils, the most dominant immune cells, play a complex and significant role in cancer initiation, progression and metastasis (1, 11, 98, 106). The N1 and N2 polarization of tumor-associated neutrophils (TANs) can be induced by IFN I and TGF-β, respectively (106). Tumor-released molecules drive a shift of infiltrating neutrophils toward an antitumor phenotype (98, 106). Neutrophils dominate the early phase of inflammation and pave the way for tissue damage to be repair by macrophages (98, 106). These functions are regulated by various cytokines and the production of their receptors (98, 106). TANs may inhibit antitumor immunity by secreting a variety of proinflammatory and immunosuppressive mediators, such as IL-1β, IL-17, TNF-α, TGF-β, VEGF, CCL4, matrix metallo-peptidase (MMP)-9, C-X-C motif chemokine ligand 8 (CXCL8) and angiopoletin-1 (ANG1) (106). Tumor-derived TGF-β promotes the accumulation of N2 neutrophils which then contribute to the formation of immunosuppressive and inflammatory TME (98, 106). Aberrant expression of TGF-β has been found in many tumor types, such as HCC, breast and colon cancer. TGF-β inhibits tumor cell growth and proliferation in the early stage, while promoting cancer cell proliferation, growth, invasion, and angiogenesis in more advanced stages (98, 106). N2 TAN can form NETs, which can promote carcinogenesis in the context of chronic inflammation (2–4, 96). The IL-8/CXCL8 autocrine signaling in tumor cells can induce the formation of NETs (98).

Dendritic cells (DCs) are antigen-presenting cells (APCs) that play crucial roles in bridging innate and adaptive immune responses (11, 106). DCs are considered main components of the TME and can promote anti-tumor T cell response. However, an immunosuppressive TME can affect DC effector functions, altering DC phenotype and promoting dysfunction and toleragenicity (106). In the TME, tumor-infiltrating DCs are frequently suppressed by tumor cells, leading to T cell tolerance rather than anti-tumor immune response (11, 35, 98). Tumor-derived factors trigger inflammation that promotes tumor growth by regulating the maturation of DCs (98, 106). For example, tumor-derived IL-6 and M-CSF convert immature DCs into macrophages and prevent the priming of tumor-specific T cells. Additionally, PD-L1 and PD-L2 expressed on DCs may also suppress the proliferation and cytokine expression of activated T cells (11, 98, 107)). Immunogenic cell death (ICD) is a unique pattern of programmed cell death that begins with the induction of cellular stress and results in cell death through the active secretion of DAMPs (108). During ICD, DAMPs interact with PRR produced by immune cells, particularly DCs, to activate innate and adaptive immune responses. ICD may provide novel strategy to increase the effectiveness of anticancer treatment since chronic exposure to TME-associated DAMPs may favor the activation of long-lasting anti-tumor immunity (98, 108). The role of DCs in the immune response induced by cancer cells undergoing ICD has been demonstrated in many studies (108). The findings indicate that the ability of different ICD inducers to initiate an efficient anti-tumor T cell response may depend DC activation in the TME (108). DCs, an important member of the innate immune system, have been reported to control the development of CAC through the production of IL-22BP, which neutralizes IL-22 (13, 14). However, cancer cells can hijack DCs to promote chronic inflammation and mitigate TAA presentation, thus accelerating carcinogenesis (13, 14, 98, 107). Different DC subsets have distinct mobilization capacities and exhibit different immunological functions (7, 8, 18, 99, 100). CCR7 is the most important chemotactic mediator of DC migration which recognizes the chemokine ligands CCL19 and CCL21 and it is the main guide in the migration of DCs to lymphoid tissue (107). The presence of inflammatory cells in some tumors, such as eosinophils in CRC and TAMs in breast and pancreatic cancer, is associated with a favorable prognosis (98, 107).

**Figure 2:**
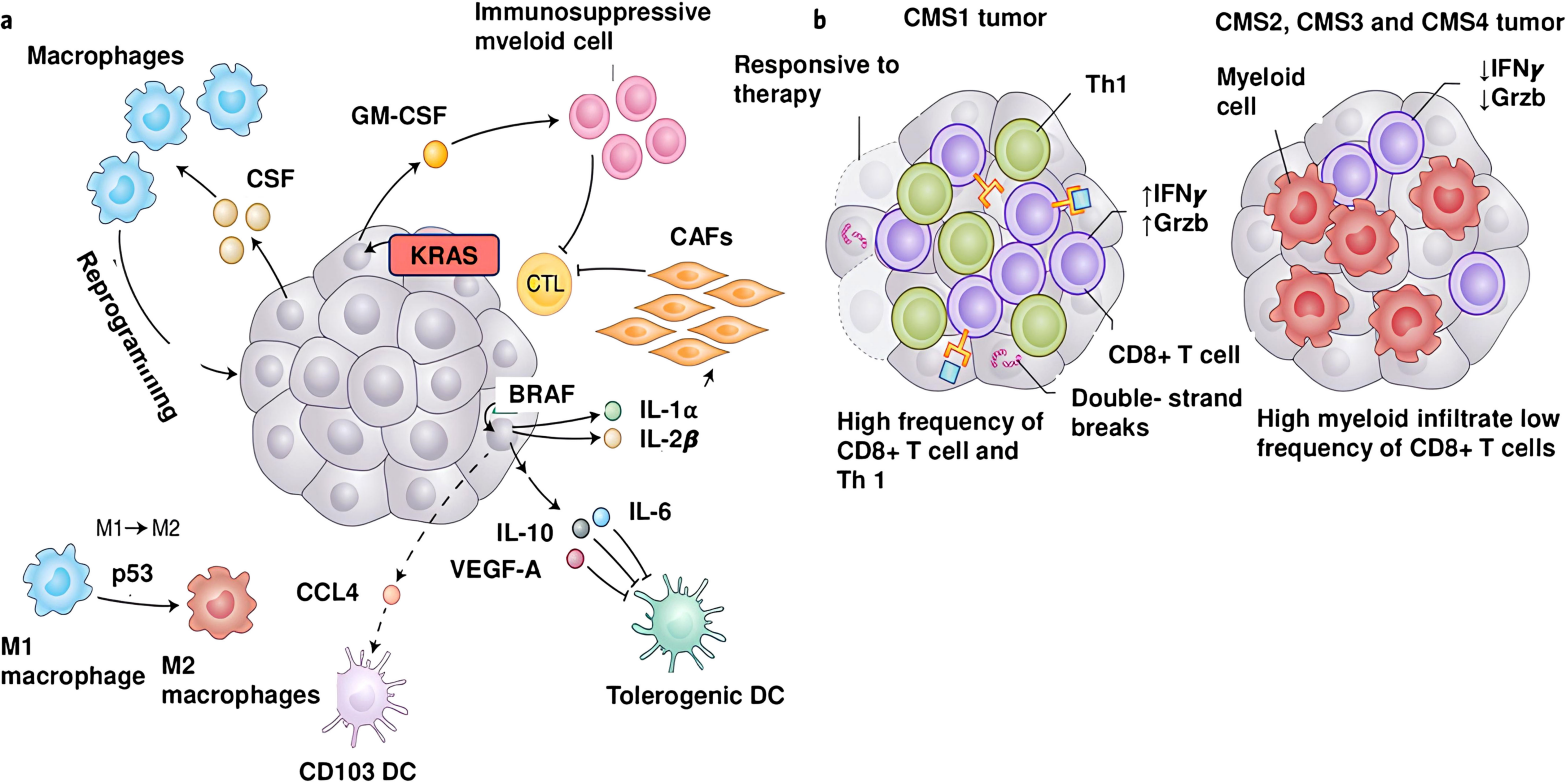
Genetic aberrations and molecules generating the inflammatory tumor microenvironment. **(a)** Oncogenes and aberrant signaling signals are key players in the development of inflammatory TME, leading to the production of inflammatory cytokines and chemokines. BRAF^V600D^ activates Wnt/β-catenin signaling, which in turn decreases production of CCL4, a chemokine important for the recruitment of CD103^+^ DCs. Additionally, BRAF^V600D^ evokes the production of IL-10 and IL-1α molecules, leading to tolerogenic DCs and CAFs in the TME. The KRAS^G12D^ mutation induces GM-CSF expression, which leads to accumulation of immunosuppressive CD11b^+^ myeloid cells in the TME. Inactivation of p53 activates signaling pathways that lead to polarization of the immunoactivating M1 phenotype to the immunosuppressive M2 phenotype. Many tumors secrete high levels of the monocyte/macrophage-promoting cytokine CSF-1. **(b)** A high mutational burden is associated with potent expression of tumor neoantigens and extensive infiltration of CD8^+^ T cells into the TME.

Myeloid-Derived Suppressor Cells (MDSCs): MDSCs are pathologically activated neutrophils and monocytes with potent immune suppressive activity (11, 98). These cells play an important role in accelerating tumor progression and undermining the efficacy of anti-cancer therapies (11, 98). MDSCs are divided into monocyte-myeloid-derived suppressor cells (M-MDSCs) with surface expression of CD11b^+^ Ly6G^+^ Ly6C^-^ high and polymorphonuclear-myeloid-derived suppressor cells (PMN-MDSCs) with CD11b^+^ LyG6^+^ LyG6^-^ low (98). The majority of MDSCs are PMN-MDSCs, accounting for more than 75%, with M-MDSCs accounting for only 10-20% (11, 98, 109). M-MDSCs have a greater capacity for immunosuppression than PMN-MDSCs (109). While PMN-MDSCs use ROS and arginase 1 (ARG1) to mediate immunosuppression, M-MDSC-mediated inhibition relies on nitric oxide (NO) and the suppressive cytokines IL-10 and TGF-β (98, 109). M-MDSCs are quickly accumulated to the inflammatory TME upon exposure to chemokines such as CCL2, CCL5, CXCL8, and CXCL12 and produce multiple immunosuppressive cytokines such as ARG1, NO, TGF-β, and IL-10 (109). The upregulation of ARG1 in MDSCs causes L-arginine deprivation that results in T cell dysfunction by inhibiting T cell receptor (98, 109). Tumor-derived factors such as VEGF, IL-6, and IL-10 accumulate MDSCs which in turn secrete more VEGF via STAT3 signaling, thus augments angiogenesis (98, 109). In addition, MDSC-derived MMPs function as a secondary angiogenic signals (11, 109). Considering that high M-MDSC numbers correlate with decreased tumor-specific T cell expansion and activation, MDSCs may be used as a novel marker to predict response to immune checkpoint inhibitors (ICIs) (11, 98, 109). On the other hand, blockade of MDSC-mediated CSF/CSF-1R signaling decreased MDSCs in the TME and converted immunosuppressive MDSCs to an antitumor phenotype, suggesting that MDSCs are targeted by inhibition of this signal (109). **Figure 2**.

The core function of innate immune system is to recognize and present tumor-associated antigens (TAAs) to cytotoxic anti-tumor effectors (5, 6). Anti-tumor effector cells can kill cancer cells directly or eliminate them by sensitizing them to biological molecules such as Fas ligand, perforin or granzyme (110–112). CD8^+^ T cells are pivotal mediators in the elimination of cancer cells, which harbor distinct T cell receptors (TCRs) (21, 113). Growing evidence indicates that several key transcriptional factors (T-bet vs Bcl-6, STAT4 vs STAT3), epigenetic mechanisms (DNA methylation and histone modification) and metabolic reprogramming are involved in the differentiation of naïve T cells into effector cells (113–115). During cancer, naive CD8^+^ T cells differentiate into CD8^+^ CTLs producing a range of chemokine receptors and effector molecules (98, 115). Factors within the TME can drive CD8^+^ T cells to exhausted T cells, which account for unique cellular phenotype, heterogeneity, and functional capacity (21, 114, 115). During exhaustion, CD8^+^ T cells gradually lose expression of IL-2 and TNF-α, and cytotoxic function (116). Terminal exhausted CD8^+^ T cells also lose IFN-α expression (116). Experimental trials have reported that terminal exhausted CD8^+^ T cells can maintain their capacity to produce molecules such as MIP1α, MIP1β, RANTES, and IL-10 (116). Immune checkpoint molecule expression of terminal exhausted CD8^+^ T cells is high and response to immunotherapies is quite low in these patients (110–112). B cells can inhibit carcinogenesis by expressing tumor-reactive antibodies, promoting tumor killing by NK cells, phagocytosis by macrophages and priming cytotoxic effector cells (111, 112). Tumor-infiltrating-B-lymphocytes (TIL-B) exert cytotoxic effect on HCC cells by producing granzyme and TRAIL (117). The majority of studies investigating the functions of TIL-B cells report that CD20^+^ TIL-B cells have positive prognostic effects (115). The prognostic power of TIL-B cells is compatible with the density of CD3^+^ and CD8^+^ T cells and T cells exert stronger anti-tumor activity in the presence of TIL-B cells (7, 8, 14, 98, 117). **Table 1**.

**Table 1:** Inflammatory cells that exhibit protumor or antitumor activity in cancer.

Cell type	Protumor activation	Antitumor activation
Dendritic cells (DCs)		
	-Promote T cell tolerance	-Provide signal for CD8^+^ cells
	-Suppress proliferation and cytokine production T cells by secreting PD-L1, PD-L2	-Support anti-tumor T cell response caused by immunogenic cell death -Prevent carcinogenesis by the production of IL-22B
Tumor-associated macrophages (TAMs)		
	-M2 TAMs cause angiogenesis by upregulation of VEGF - degradate ECM - activate the response of endothelial cell to growth F - upregulate TGFβ that promotes EMT	- M1 TAMs promote anti-tumor activities of cytotoxic CD8^+^ T cells and NK cells
Tumor-associated neutrophils (TANs)		
	-Induce tumor angiogenesis by promoting release of VEGF -Inhibit anti-tumor immunity by the expression proinflammatory mediators -Generate immunosuppressive TME -Promote the remodeling of TME that induces tumor cell extravasation	-N1 TANs display an anti-tumor activity by direct or indirect cytotoxicity
Myeloid-derived suppressor cells (MDSCs)		
	-Inhibit anti-tumor immunity by secreting immunosuppressive molecules -Induce tumor angiogenesis via VEGF and matrix metallopeptidase -Decrease the proliferation and activation of tumor-specific T cells by production colony-stimulating factor-1 receptor	
Vascular endothelial cells		
	-Induce migration of tumor cells due to weakened vascular endothelial junctions upon inflammation	-Form a barrier for blood components including tumor cells to infiltrate tissues under physiological conditions

### 4.4 Main inflammatory pathways in cancer

#### 4.4.1 Cyclooxygenase (COX) signaling

Chronic inflammation triggers the production of inflammatory mediators and activates signaling pathways that promote tumor growth, metastasis, and angiogenesis (11, 98). Among these mediators, prostaglandins (PGs) play a crucial role in the initiation and progression of inflammation and cancer (118). The expression of PGs is regulated by COX, which consists of three isoenzymes, namely COX-1, COX-2 and COX-3 (118). Recent studies have shown that COX-2 is upregulated in many tumors, including CRC, HCC, breast, PDAC and gastric cancer and COX-2 overexpression is associated with an unfavorable prognosis (119). Among the 5 key PGs derived via COX pathway, PGE2 is the most important PG in cancer and its upregulation is associated with advanced tumor stage. PGE2 orchestrates IFN-γ synthesis of NK cells, which is a significant inflammatory process (98, 119). PGE2 potentiates M-MDSCs and impairs the proliferation capacity of T cells (98, 119, 120). Furthermore, tumor-derived PGE2 induces NF-κB which epigenetically reprograms monocytes toward an immunosuppressive phenotype (11, 118–120). PGD2, another COX-2 metabolite, may play dual roles in in chronic inflammation and cancer. PGD2 can promote or inhibit tumor cell growth and metastasis depending on the stage of the tumor (119, 120). COX2/PGE2 signaling promotes the accumulation of MDSCs, leading to a decrease in the number of activated CD8^+^ T cells in the TME (98, 118, 120). PGE2 also affects the polarization of macrophage by triggering monocyte differentiation into the M2-TAMs (98). Considering the contribution of the COX2/PGE2 pathway to the formation of immunosuppressive TME, inhibiting this pathway may increase the effectiveness of immunotherapies (11, 118–120).

#### 4.4.2 Lipoxygenase (LOX) signaling

The LOX signaling mainly includes 5-LOX, 12-LOX, and 15-LOX (11). While 5-LOX ve 12-LOX display angiogenic and protumorigenic activity, 15-LOX exhibits both protumorigenic and antitumorigenic impacts (98, 121, 122). Given that 5-LOX and COX2 are upregulated in inflammation-associated tumors, suppression of these two molecules together may lead to more potent tumor suppression (98). The 12-LOX enzyme induces the forming of 12-HETE which promotes tumor growth by activating the integrin-linked kinase/NF-κB pathway (121). 15-LOX-1 may be secreted in Hodgkin lymphoma cells, and its metabolites boosts tumor-associated inflammation (121, 122). A recent study has indicated that 15-LOX levels are lower in doxorubicin (DOX)-resistant cells than DOX-sensitive cells (123). The overexpression of 15-LOX may trigger DOX recruitment in DOX-resistance cancer cells and induce their apoptosis (123). An experimental study demonstrated that 15-LOX promotes the resolution of inflammation in lymphedema developing in breast cancer mouse models by controlling Treg via IFN-β (122). The LOX pathways regulate the metabolism of arachidonic acid to leukotrienes such as leukotriene A4 (LTA4) and leukotriene B4 (LTB4) (121). 5-LOX is a central enzyme in LT biosynthesis, a potent arachidonic acid-derived lipid mediators released by innate immune cells, that control inflammatory processes (121). In addition, the enzyme is involved in the generation of omega-3 fatty acid-based oxylipins which promote the resolution of inflammation (121). LTB4 promotes the progression of inflammatory-derived tumors and inhibition of the LTBB4 receptor can suppress the progression of these tumors (121). The leukotriene D4 (LTD4) is upregulated in patients with HCC and chronic hepatitis B (121). Currently, the benefits of combining leukotriene receptor antagonists with multi-kinase inhibitors in the treatment of HCC are being investigated in many studies (98, 121).

#### 4.4.3 JAK-STAT Signaling Pathway

The Janus kinase (JAK) signal transducer and activator of transcription (JAK-STAT) pathway is an evolutionary conserved signaling pathway that functions in several crucial physiological processes, including hematopoiesis, differentiation, metabolism, and inflammation (124, 125). The JAK protein family contains four members: JAK1, JAK2, JAK3, and TYK2. The STAT family involves seven members: STAT1, STAT2, STAT3, STAT4, STAT5A, STAT5B, and STAT6 (124, 125). More than 50 types of cytokines, including IFNs, ILs, and growth factors, have been indicated to play roles in JAK-STAT signaling to fulfill regulatory functions in cell differentiation, metabolism, survival, homeostasis, and immune response (124, 125). Once receptors bind to an extracellular ligand, JAKs induce tyrosine phosphorylation of the receptors and accumulate corresponding STATs. The phosphorylated STATs then dimerize and enter the nucleus to orchestrate specific gene transcription (124, 126). STAT3, the core member of the STAT protein family, plays multifaced roles in the inflammatory responses and tumor progression (124, 126). The dysregulated STAT3 pathway has been indicated to play a role in the development of many inflammatory diseases such as rheumatoid arthritis, and IBD (124–126). Furthermore, persistent activation of STAT3 signaling can induces carcinogenesis (126). Cytokines promoting the activation of STAT3 are upregulated in IBD, such as IL-1β, IL-6, IL-12, IFN, and TNF-α (124). The IL-6/STAT3 pathway, a critical regulator of the inflammatory process, plays a role in the pathogenesis of CAC (124, 126). In addition, CAFs secrete IL-6 which upregulates the production of metastasis-associated markers such as leucine Rich Alpha-Glycoprotein 1 (LRG1) via the JAK/STAT3 signaling (98, 126). In CRC, disruption of the intestinal epithelial barrier integrity and sensing of PAMPs by PRR activates the STAT3 pathway, thereby initiating the inflammatory response (124, 126).

#### 4.4.4 Non-coding small RNA

Over the last two decades, many studies have highlighted the functional and therapeutic relevance of small non-coding microRNAs (miRNAs) in inflammation and cancer (127, 128). Quite recently, a new class of such RNAs have been identified, termed circular RNAs (circRNAs), that have been identified to play critical role in inflammatory diseases (127). The physiological and pathological functions of circRNAs occur through miRNA sponging, interaction with circRNA-binding proteins (cRBPs), protein-translation, or transcriptional regulation. circRNA regulate tumor-associated inflammatory signaling pathways mainly through miRNA sponging, circRNA-binding proteins (cRBBP) binding, and protein translation (127). The signaling pathways, such as Akt, E-cadherin, EGFR, MAPK, NF-κB, STAT, TGF-β, VEGF, and Wnt/β-catenin have been found to involved in tumor-associated inflammation (127, 128). NF-κB is involved in recruiting inflammatory cells and mediating the release of inflammatory chemokines, thus creating an inflammatory TME that promotes tumor progression (11). circRNA can form circRNA-protein complex to modulate signaling pathways in tumor-associated inflammation (127). Some of the proteins encoded by circRNA participate in signaling pathways to modulate the development and progression of inflammation and cancer (127, 128). Many studies have indicated that circRNAs regulate DC- and neutrophil-mediated immune response, as well as the activation of TAN, TAM, and CAF (127). Inflammasome formation is the relevant mechanism that drives inflammation in immune cells by activating cysteine protease caspase-1, which subsequently induces pyroptosis through the secretion of inflammatory cytokines. circRNAs may play a role in tumor-mediated regulation of the immune system (127).

## 5 Cancer therapy-induced inflammation

Anti-cancer therapy is associated with an inflammatory response in tumor tissue, which either drives an anti-tumor immune response or, conversely, promotes tumor growth (129). Recent studies have demonstrated the impact of cancer therapy-induced inflammation on both tumor recurrence and clinical outcomes (129–132). Death of cancer cells elicits an anti-tumor immune response and that lipid mediators such as prostaglandin 2 (PG2) and platelet activation factor (PAF), play a role in the clearance of dead cells in the TME (129, 132, 133). Anti-cancer treatments are very effective in destroying cancer cells, but the main challenge in cancer treatment is that some cancer cells cannot be eliminated and they proliferate, causing tumor recurrence (132–134). Chemotherapy and radiotherapy often cause apoptotic cell death (129, 131, 132). In fact, apoptosis is a physiological process in which apoptotic cells are cleared by phagocytes such as macrophages and DCs, which prevents the emergence of inflammation by inhibiting the release of proinflammatory cytokines such as IL-10 and TGF-β, from phagocytes (102, 103). However, during the anticancer therapy, delayed clearance of apoptotic cells can lead to secondary necrosis, which can result in the release of proinflammatory cytokines and thus inflammation (103, 132).

Another type of cell death caused by anti-cancer treatments, including chemotherapy and radiotherapy, is necrosis (129, 132–134). The main landscape of necrotic cell death is the rupture of the cancer cell membrane and the release of DAMPs, which engage distinct receptors present on the innate immune cells (129, 132, 133). Upon recognition, DAMPs activate DCs and promotes the engulfment of dying cells, thereby improving anti-tumor response and clinical consequences (129, 132, 134). Conversely, DAMPs released from dying cancer cells accumulate macrophages to promote cancer cell clearance and polarize them to a M2 phenotype, which may contribute to immunosuppression and cancer cell resistance to anti-cancer therapy (102, 103). Dying cancer cells also express lipid mediators, such as PGE2 and PAF, promoting the proliferation and survival of remaining cancer cells (102, 103, 129, 132). Experimental trials have revealed that DAMPs are not only released from necrotic cells, but certain forms of programmed cell death can induce DAMPs release and lead to immunogenic cell death (129, 132, 134). Distinct chemotherapeutic agents, such as anthracyclines, oxaliplatin, cyclophosphamide, mitochontrone, bortezomid and radiotherapy result in immunogenic cell death (ICD) of cancer cells, and stimulate the release of DAMPs from dying cells (129–133). Following ICD, DAMPs promote the activation of cytotoxic effectors, which play a crucial role in the therapy response (130–132). Chemotherapeutic agents exert anti-cancer influences by influencing main cellular biological processes, which are indispensable for the robust proliferation of tumor cells (132). In addition, anti-cancer drugs modulate the immune cell profile of the TME, by triggering immune reactions and promoting cytotoxic effectors (135, 136). A few anti-cancer agents, such as cisplatin, can cause chronic inflammation through the release of some pro-inflammatory mediators, leading to angiogenesis, tumor progression and resistance to treatment (136, 137).

Currently, radiotherapy has established an important part of conventional cancer treatments, as high dose of radiation kills cancer cells and reduces tumor size (138, 139). Radiotherapy activates transcription factors, including NF-κB and STAT, which generate multiple radioresistance signals through modulating anti-apoptotic pathways (138, 139). Radiotherapy-induced NF-κB activation contributes to the prevention of apoptosis and cell cycle arrest (139) Additionally, NF-κB activation also orchestrate the transcription of a myriad of genes regulating immunity, proliferation, invasion, and angiogenesis, which favor radiotherapy resistance (133, 138, 139). Experimental trials in some types of cancer have reported that drugs that suppress the biological effects of NF-κB, such as indomethacin and curcimin, enhance the radiosensitivity of cancer cells by accentuating radiotherapy-induced apoptosis (133, 139). Irradiated tumor cells secrete many soluble molecules, including proinflammatory cytokines, that also have biological effects on non-irradiated cells (138, 139). In addition, radiotherapy promotes the formation of a proinflammatory TME, by inducing the network between inflammatory pathways, ultimately leading to tumor cell death (133, 139). Radiation-induced inflammation accentuates the adaptive antigen-specific immune response and tumor-host interaction is reshaped during radiotherapy, which contributes to the favorable clinical outcomes (133, 139). Additionally, chronic inflammation induced by radiotherapy in the TME promotes to recruitment of immunosuppressive cells (TAMs, myeloid-derived cells, and regulatory T cells) (133, 139). Radio-therapy can foster proliferation of cancer cell and tumor progression, thus paving the way for treatment resistance (133, 139).

**Figure 3:**
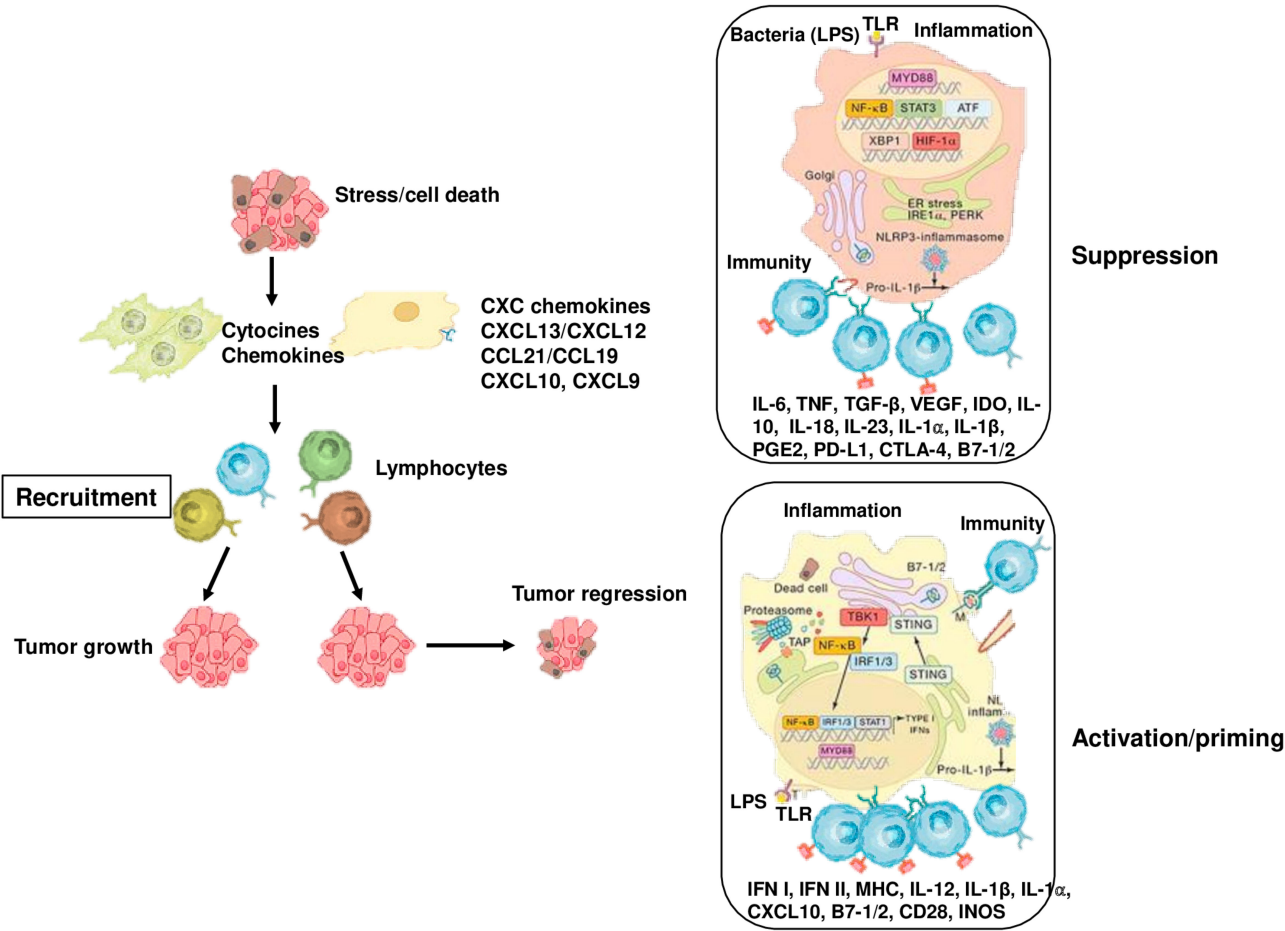
Cancer-associated inflammation that affects tumor growth and progression. Stress, cell death, obesity and bacterial infection and its components trigger the activation of innate immune cells and increase the expression of inflammatory mediators, which induce the recruitment of adaptive immune cells to damaged tissue. Myeloid cells, such as DCs, take up antigens and present them T cells, activating to CD8^+^ T cells. On the other hand, cell death may exert immunosuppressive and tolerogenic effects, thus inhibiting CD8+ T cell activation. Similarly, monocytes and macrophages can impede the anti-tumor activity of CD8+ T cells by producing IL-10, ARG1, IDO, and TGF. In the early stages of cancer, inflammation leads to the production of cytokines, such as IL-1, TNF, and IL-6, that promote tumor growth, as well as VEGF that supports neo-angiogenesis.

In some solid tumors, such as HCC, immune checkpoint inhibitors (ICIs) have been shown to significantly improve overall survival (140, 141). However, immune-related tissue injury is common in patients with cancer receiving ICIs, adversely affecting clinical outcomes (141, 142). Although immune-related tissue injury can affect any organ, it is most commonly observed in the skin, endocrine system, gastrointestinal system and liver (142). In randomized controlled trials (RCTs), the incidence of immune-related tissue injury has been reported to be between 1% and 15% (141, 142). In particular, in patients with HCC receiving ICIs, the incidence of immune-related liver damage is directly related to the degree of underlying liver disease and the combined administration of molecularly targeted drugs (141–143). ICIs may cause inflammation due to lysis of tumor cells, especially in patients who achieve an objective radiological response (142, 143). ICIs also led to increased T cell infiltration within the TME, resulting in overproduction of proinflammatory cytokines by these cells, thereby enhancing the inflammatory response (141–143). Cytokine release syndrome (CRS) is one of the most serious clinical toxicities of immunotherapies (143). Many studies have shown that CRS can develop not only during the use of immunotherapies in patients with cancer but also during the treatment of other dis-eases, where CRS is accompanied by severe inflammation (143). Recently, two prospective studies of patients with advanced HCC who received ICIs reported that patients with high baseline AFP levels had a higher frequency of immune-related liver injury and significantly higher levels of inflammation markers than patients with normal AFP levels (142). Although ICIs have provided significant progress in cancer treatment, they fail in a relevant proportion of cancer patients due to their side effects and the development of treatment resistance (142, 143). The main reason for resistance to immunotherapies is the inflammatory TME, where chronic inflammation is the fundamental driver of cancer cell proliferation, angiogenesis, and recruitment of immunosuppressive cell populations (141–143) **Figure 3**.

## 6 Targeting tumor-associated inflammation: leveraging precision medicine

Tumor-associated inflammation, which contains complex crosstalk between epithelial and stromal cells, can lead to epigenetic alterations that drive malignant progression and even initiate tumorigenesis (1, 11, 35). In addition, chronic inflammation triggers the production of growth factors that support the newly emergent tumor (98, 144, 145). Additionally, tumor-associated inflammation alters the effectiveness of anti-cancer drugs through modulation of the production of multidrug efflux transporters (e.g., ABCG2, ABCB1, and ABCC1) and drug-metabolizing enzymes (e.g., CYP1A2 and CYP3A4) (146). Furthermore, inflammation can protect cancer cells from drug-mediated cell death by regulating DNA damage repair, downstream adaptive response (e.g., apoptosis, autophagy, and oncogenic bypass signaling), and TME (144, 146). Inflammation-reducing chemopreventive treatments that inhibit either the initiation or propagation of persistent inflammation may therefore prevent or delay cancer (98, 144, 146). Many studies have reported that anti-infective agents, non-steroidal anti-inflammatory drugs (NSAIDs), and other drugs capable of reducing inflammation, such as statins and metformin, reduce the risk and incidence of cancer (98, 144).

### 6.1 Non-steroidal anti-inflammatory drugs (NSAIDs).

COX2/PGE2 signaling pathway can provide epithelial cells with protumorigenic properties, thereby promoting the development of inflammation-related cancer (98, 144, 146). COX2 can activate the AKT, mTOR, and NF-κB pathways to support cancer cell proliferation either directly or via PGE2 signaling (144, 146). Aspirin and selective COX-2 inhibitors have pro-apoptotic and antiproliferative effects on COX2- overexpressing cancer cells (98). In addition, PGE2 silences TSGs by reinforcing their promoter methylation through a EP4-DNA methytransferase pathway (146). In mice, celexoxib treatment limits intestinal tumor growth and can reverse the silencing of TSGs and activation of oncogenes in COX2 overexpression-induced HCC (144). NSAID treatments prevent colorectal carcinogenesis by blocking the senescence-associated inflammatory response. PEG2 evokes colorectal carcinogenesis via YAP1, a transcriptional regulator in mice, and administration of NSAID indomethacin impedes colon tumorigenesis (146).

NSAIDS exhibit their anti-inflammatory effects by inhibiting cyclooxygenase activity (98). Aspirin can inhibit the nuclear translocation of NF-κB, thereby inhibiting the P13 kinase/Akt-mediated cell survival pathway and promoting cell apoptosis (144, 146, 147). In some types of cancer, aspirin inhibits tumor cell proliferation and evokes apoptosis by upregulating Bax and downregulating Bcl-2, changing the ratio of Bax/Bcl-2 (98). In a systematic review and meta-analysis of observational studies, the risk of CRC was found to be 27% lower in individuals who regularly used aspirin compared to non-users, the risk of esophageal and gastric cardia cancer was 39% lower, the risk of stomach cancer was 36% lower, the risk of hepatobiliary cancer was 38% lower, and the risk of PDAC was 22% lower (148). However, some studies reported that aspirin use did not reduce the risk of PDAC (144). Additionally, studies conducted in Sweden and Taiwan revealed that aspirin use reduce the risk of HCC in patients with chronic viral hepatitis (149, 150). Post-diagnostic aspirin administration reduces overall mortality in GI tumors, and the survival benefit is better in PIK3CA-mutant and COX-2-positive tumors or tumors with low PD-L1 levels (151). As a selective COX-2 inhibitor that inhibits prostaglandin production, celecoxib can induce apoptosis by activating transcriptional regulators of ER stress in hepatoma cells (152). Dexamethasone can evoke apoptosis in multiple myeloma mediated by miR-125b expression (144).

### 6.2 Antiviral therapies

In addition to the direct oncogenic effects, HBV and HCV infections can cause cancer-promoting inflammation. In infected individuals, antiviral therapies that inhibit HBV and HCv replication, such as interferon-based therapies, nucleoside or nucleotide analogues, and direct antiviral agents, reduce the risk of HCC by 50-80% (153). Antiviral therapies are also effective in reducing disease recurrence and improving postoperative survival outcomes in patients with HCC (153). Similarly, more than 90% of cervical cancers are associated with human papillomavirus (HPV). Many RCTs have indicated that HPV vaccines are highly effective in preventing cervical precancerous lesions (154). Today, HPV vaccination has been implementing for cervical cancer prophylaxis in multiple age groups across different countries (154). Unfortunately, drugs to treat Epstein-Barr virus (EBV), the first tumor virus identified in humans and associated with stomach cancer and lymphoma, have not yet been identified (144). Helicobacter pylori is the strongest risk factor for gastric cancer, and its eradication with broad-spectrum antibiotics not only prevents gastric cancer, but also reduces the rate of developing metachronous gastric cancer in patients with early gastric cancer (17).

### 6.3 Cytokine- and chemokine-directed therapies.

The elimination of MDSCs in the TME by inhibiting IL-1 pathway is a potential strategy to overcome tumor resistance to immunotherapies (144). Anti-IL-1β monoclonal antibodies (mAbs) can boost the efficacy of PD-L1 inhibition in some types of cancer (144). In CANTOS study, canakinumab, an anti-IL-1β mAb, dose-dependent reduced IL-6 and C-reactive protein (CRP) in atherosclerotic patients with prior myocardial infection and elevated CRP levels (155). IL-2 is a key growth factor for CD4^+^ T cells and NK cells, which displays antitumor activity. IL-2 treatment can confer durable response in melanoma and renal cell carcinoma (RCC) patients (98). IL-6 is one of the most pivotal cytokines linking cancer-promoting inflammation and immunosuppression (144). Drugs targeting IL-6 exhibit limited activity in patients with cancer when used as monotherapy (144). Galunisertib, a small molecule inhibitor of the TGFβR1 kinase, has been indicated to be safe in patients with various cancers (156). Galunisertib provides modest therapeutic activity when combined with gemcitabine in patients with unresectable PDAC or with sorafenib in advanced HCC (156). Monoclonal antibody therapies targeting TNF have been applied to patients with advanced stage, and modest therapeutic effects have been observed (157).

Can precision medicine contribute to the management of tumor- associated inflammation? Each patient has a unique genome, proteome, epigenome, microbiome, lifestyle, diet, and other characteristics that all interact to influence oncogenes, disease progression, effective treatment options, drug tolerance, remission, and recurrence (145). Molecular characterization of tumors has shifted cancer treatment strategies away from nonspecific cytotoxic treatment of histology-specific tumors toward targeting of actionable mutations that can be found across multiple cancer types (145). Precision oncology provides individualized treatment of cancer on a per-patient basis, based on the unique DNA fingerprint of a patient’s cancer (145). Recent studies have identified prognostic and predictive molecular markers that could improve diagnosis, treatment planning, and clinical outcomes (158–165). However, as highlighted in detail in this review article, although the inflammatory cells, soluble molecules, genetic and epigenetic mechanisms involved in the formation of tumor-associated inflammation have been largely elucidated, there is no molecular marker for tumor-associated inflammation yet used in clinical practice.

## 7 Conclusions and future perspectives

In recent years, technological advances, including single-cell multi-omics and spatial technologies, artificial intelligence-based systems biology approaches have led to a breakthrough in our knowledge of how cancer cell-intrinsic features regulate immune cell composition, spatial distribution and functional status of the TME. These tremendous advances have allowed us to understand more about the interactions between cancer cells, immune cells and stromal cells. Chronic inflammation is thought to be a fundamental feature of cancer, which plays a key role in establishing TME. TME consists of cancer cells, immune cells, stromal cells, ECM proteins, intratumor microbioma and soluble molecules, such as proinflammatory cytokines, chemokines, growth factors and immune checkpoint molecules. TME determines the biological behavior of cancer cells and is therefore an important driver in tumor progression, suppression of antitumor immunity and resistance to anticancer therapy. The immune cell composition, activation status, and spatial distribution of the TME vary among tumor types. Cancer cell molecular characteristics, including genetic and epigenetic alterations, signaling pathway deregulation and altered metabolism play a critical role in governing the composition and functional status of immunological landscape and affect the efficacy of immunomodulatory therapies. Immune cells, like cancer cells, have multifaced functions in the TME, their activities ranging from protumorigenic to antitumorigenic.

Inflammation in the tumor tissue can be induced at different times and stages, i.e. before or after carcinogenesis. In cancer, inflammatory pathways that evolved to elicit immunity against infection and maintain tissue homeostasis are hijacked by cancer cells. Chronic inflammation acts as a tumor promoter by boosting cell survival, proliferation, and angiogenesis during tumorigenesis and tumor progression. In addition, chronic inflammation is involved in carcinogenesis by activating NF-KB and STAT signaling pathways. In the vast majority of tumors, inflammation exerts dual effects; normally, immune cells inhibit tumor growth by eradicating tumor cells, on the other hand, in inflammatory tumors, some immune cells, inflammatory cells and molecules promote cancer growth by boosting cancer cell proliferation and prolonging its survival. A significant number of cancer patients receiving ICIs emerge primary or acquired drug resistance. The interaction between cancer cells and stromal cells in the TME can alter the cellular composition and soluble molecule landscape of the TME and may predispose to the development of drug resistance. Therefore, it is necessary to elucidate this complex crosstalk between cancer cells and immune cells, particularly inflammatory cells to develop effective anti-cancer drugs and improve the overall survival of cancer patients. Considering all of these advances, it can be suggested that treatment of cancer-associated inflammation, elimination of immunosuppressive landscape of the TME and repolarization of terminal exhausted CD8+ T cells into effector CD8+ T cells are necessary for the anti-cancer therapies to be successful in GI cancers. Cancer cell molecular features can be therapeutic targeted to transform the cancer-promoting immune landscape into a tumor-suppressing immune landscape.
